# Long rallies and next rally performances in elite men’s and women’s badminton

**DOI:** 10.1371/journal.pone.0229604

**Published:** 2020-03-03

**Authors:** Miguel A. Gomez, Anthony S. Leicht, Fernando Rivas, Philip Furley

**Affiliations:** 1 Faculty of Physical Activity and Sports Sciences, Department of Social Sciences, Physical Activity, Sport and Leisure, Polytechnic University of Madrid, Madrid, Spain; 2 Sport and Exercise Science, James Cook University, Townsville, Queensland, Australia; 3 Spanish Badminton Federation, Madrid, Spain; 4 Institute of Training and Computer Science in Sport, German Sport University Cologne, Cologne, Germany; Universidade de Tras-os-Montes e Alto Douro, PORTUGAL

## Abstract

The aim of the present study was twofold: (i) to identify contextual variables associated with the occurrence of long rallies while investigating time-related and technical parameters; and (ii) to identify performance differences between long rallies and the subsequent rally when accounting for match-context and the players’ sex. The sample included 60 men’s (n = 4,475 rallies) and 60 women’s (n = 4,490 rallies) matches randomly selected from the 2015 World Badminton Super Series and World Championship (the final sample included long rallies that had an immediate next point played: n = 1,734 and n = 1,644 rallies for male and female players, respectively). The long rallies represented 19.4% (n = 867) and 16.5% (n = 822) of total rallies for male and female players, respectively. Long rallies were established using a two-step cluster model based on rally time and number of strokes for male (13-79s, 14–72 strokes) and female players (11-56s, 11–52 strokes). The variables collected were point outcome (when serving and receiving, winner, forced-error and unforced-error), number of strokes per rally, rally time, rest time, density, and time between strokes. The rallies were classified into different contexts (clusters) according to influencing factors with eight clusters for male players and three clusters for female players identified. Comparisons among clusters were conducted using Kruskal Wallis and one-way ANOVAs. Comparisons between long and immediate next points were conducted using the Wilcoxon tests for most variables and Crosstabs Command for point outcome and rallies (long and immediate next). Statistically significant differences were identified for both sexes among clusters only for time-related variables (i.e., rally time, rest time, density and time between strokes). In addition, a greater number of strokes, longer rally, rest time, and higher density were identified during long rallies compared with the immediate next rally for both men’s and women’s matches (p<0.05). The time between strokes during long rallies was significantly greater for male players during clusters 3, 5, 6, and 7 (p<0.05) and significantly lower for female players during all clusters (p<0.05). Significant relationships were identified between winning point outcome, and more unforced errors when serving during the immediate next rally (men’s cluster 5 and women’s cluster 2), and more winners when serving during the immediate next rally (men’s cluster 6). The current study identified and characterised long rallies in elite men´s and women´s badminton matches highlighting the importance of sex and contextual factors on time-related and technical demands. Information obtained from these unique sequences of play (i.e., long and immediate next rallies) will assist coaches when modelling and simulating players’ performances (i.e., physiologically and cognitively) during athlete preparation/competition.

## Introduction

Prior research in elite badminton has primarily focused on physiological, anthropometrical, biomechanical, visual fitness, anticipatory skill, and temporal and notational analyses [1]. In particular, performance analysis has been widely used to understand the time-related factors of the game (e.g., rally duration, resting times, frequencies between strokes or density of play) that allow for designing training scenarios and drills that simulate the competitive requirements and the high intensity actions of badminton rallies [1–3]. Chiminazzo, Barreira, Luz, Saraiva, and Cayres [4] reported that a badminton match consisted of high-intensity and short-duration intermittent efforts, interspersed by brief periods of rest. During rallies, numerous motor actions including racquet strokes occur as a result of player displacements and tactical actions (Seth, 2016). These technical, tactical, and timing variables have been the focus of investigation during badminton matches [1], showing, perhaps not surprisingly, that longer rallies coincided with greater rest time, and ultimately longer matches [5]. The inclusion of longer rallies with a greater number of quicker strokes per rally result in a more challenging physical and technical demanding performance during matches [5]. Further, Abian-Vicen, Castanedo, Abian, and Sampedro [6] argued that the highly demanding structure (increased frequency of strokes per second) and heightened work density of rallies reduced the decision time between points and placed greater cognitive load on players during badminton matches.

More recently, Laffaye et al. [3] identified that the temporal structure of elite badminton was becoming more intense (i.e. 34% increase in stroke frequency, 0.9/s– 1.3/s and 5.5–10.2 strokes per rally; and a 34.5% increase in playing time). In fact, the rhythm of strokes and the rally duration has increased substantially during matches with an increase number of long rallies (>19s) accounting for 15% of the total number of rallies played during matches. Consequently, badminton players have to deal with different competitive scenarios (i.e., long and regular duration rallies) that impose challenging physiological and psychological demands that may affect consecutive performances during matches/games. To date, the effect of long rallies and their immediate effect on subsequent rallies/performance is largely unknown. To our knowledge, only one study has investigated this issue via observational analysis of a small sample of 17 matches during the 1996 European Badminton Circuit Tournament at Lisburn, Ireland 1996 [7]. Based upon these matches, rallies undertaken immediately after long rallies were shorter (i.e., 2-3s shorter mean rally length) mainly as a result of unforced errors [7]. The authors highlighted the importance of understanding the time-related factors of badminton matches when preparing players as they may be affected by physical and cognitive fatigue, particularly after long rallies [7]. In fact, Abian et al. [6] argued that coaches need to be aware of the different point scenarios (i.e., rally duration and timing demands) that occur during the match with sound research based on adequate sample sizes needed to assist coaches in simulating training scenarios that have the potential to mitigate debilitating effects of strenuous long rallies on subsequent rallies. During badminton matches, players are confronted with a combination of rapid decision-making, motor performances, pressure (e.g., higher under critical scenarios), and physiological arousal (e.g., higher with intermittent efforts) [8]. For example, pressure can lead to cognitive anxiety that potentially increases during highly demanding contexts such as long rallies during badminton matches given the high physical and physiological demands [9]. These greater demands may also have an impact after a long rally and contribute to debilitating performances as the match progresses. Therefore, understanding how the physical (i.e., time-related parameters of player’s workload such as rally time, rest time, density of play, and time between strokes) and cognitive demands (i.e., the correct technical-tactical decision and execution to win the rally) of competition influence elite badminton players’ performances is crucial in developing better preparatory regimes for players to succeed during competition.

Prior studies examining elite badminton game characteristics have focused on isolated match analysis [1,3,4] and did not account for time-related and technical variables, or the specific contextual variables (i.e. set number, interval of points, score-line or match duration) that result from high-intensity actions (e.g. long rallies and the immediate next rally). Specifically, little attention was given to performance variability during matches and its influence on point outcome [4]. Therefore, the present study attempted to account for these shortcomings of prior research by exploring the time-related and technical aspects of long rallies and their immediate impact on the next rally while accounting for competition context (i.e. contextual variables). More specifically, the present research sought (i) to identify the contexts in which long rallies occur while differentiating time-related and technical parameters; and (ii) to identify performance differences between long rallies and the immediate next rally played. We hypothesized that subsequent rallies played immediately after a long rally would involve lower time-related parameters (e.g., rally time, rest time, frequency, density and frequency between strokes) and more unforced errors modulated by competition contextual factors.

## Material and methods

### Sample

The sample comprised of 60 matches each for male and female players (n = 120; n = 4,475 and n = 4,490 rallies, respectively) randomly selected from the total number of matches played during the 2015 World Badminton Super Series and World Championship season (n = 449 and n = 434, for men´s and women´s respectively) that met the following criteria: (i) publicly available on TV; (ii) included all rallies played within the full match without omissions; and (iii) included both regular and playoff/knockout stage matches. To account for the disparity of match-performances in elite badminton [10], analyses accounted for match time-duration and time-related structure of rallies for each sex. Therefore, a two-step cluster analysis was conducted (split by sex) initially using number of strokes and rally time as quantitative variables to determine the final sample in accordance with the specificities of long rallies [3]. The models obtained were very good (Silhouette measures > 0.8) and identified two types of rallies played: long (24.5% of the total rallies with rally duration between 13 and 79s, and a mean of 21.1±8.2s; number of strokes between 14- and 72 strokes and a mean of 22.3±8.0 strokes for male players; and 19.6% of the total rallies played with rally duration between 11 and 56s, and a mean of 19.7±6.4s; and number of strokes between 11 and 52, and a mean of 19.4±6.1 for female players); and regular (75.5% of the total rallies played with rally duration between 0 and 12s and a mean of 6.3±3.1s; and number of strokes between 1 and 13, and a mean of 6.8±3.4 strokes for men’s players; and 80.4% of the total rallies with rally duration between 1 and 11s, and a mean of 6.5±3.1s; and number of strokes between 1 and 10, and a mean of 6.1±3.2 for women’s players) rallies. The final sample included only the long rallies that had an immediate next point played (i.e. repeated measure): n = 1,734 and n = 1,644 rallies for male and female players, respectively. The long rallies represented 19.4% (n = 867) and 16.5% (n = 822) of total rallies played for male and female players, respectively. All data were obtained from publicly available sites in accordance with the European Data Protection Law and the current study was conducted with the approval of the Polytechnic University of Madrid Ethics Committee.

### Procedure

Several initial stages were conducted to validate data and define/identify variables. Firstly, Stage 1 involved the collation of the sample where match analysis was carried out using a video analysis program (Dartfish, Friburgo, Switzerland) by four observers (Sports Scientists with ten years of experience as elite badminton coaches) trained to collect match temporal and notational variables. The inter- and intra-observer reliability for match analysis was assessed as good to very good (Kappa = >0.81; Pearson’s correlation coefficient: r>0.86; Intra-Class-Correlation coefficient, ICC = >0.85, and the typical error of measurement, TEM = <0.46) [11–12] from a selection (n = 12) of randomly selected matches.

Stage 2 included the identification of variables for analysis from matches. The main independent variable studied was long rally and the immediate next rally while the dependent variables (see definitions in Table 1) were related to the *time-related factors* of the game: rally time, rest time, density, and time between strokes; and the *technical variables*: number of strokes per rally and rally outcome.

**Table 1:** Time-related factors, technical and contextual variables analysed.

Variables	Definition
*Time-related factors*	
Rally time	Time duration in seconds from the serve stroke until the end of the rally.
Rest time	Time duration in seconds between the end of the rally and the serve action of the immediate next rally.
Density	Ratio of rest time and rally time.
Time between strokes	Time duration in seconds between strokes of both players.
*Technical variables*	
Number of strokes	Total number of strokes performed per rally.
Rally outcome	Based upon initial designation of player that wins the rally = serving or receiving; and action leading to outcome = winner, forced error or unforced error.
*Contextual variables*	
Match type	According to the total match duration (two-step cluster analysis) as regular (ranged between 25.4 and 59.5 min for men´s matches and between 21.2 and 55.8 min for women´s matches) and long matches (ranged between 76.9 and 91.0 min for men´s matches and between 70.4 and 110.6 min for women´s matches).
Match set	1^st^, 2^nd^ or 3^rd^
Set Interval	Controlling for the score played before the time-break of 1-min when one player reaches a score of 11 points = before and after reaching a score of 11 points.
Score-line	Difference in the score between players (considering the player that wins the point as the reference), based on a two-step cluster analysis considering three conditions for men’s players: winning, losing, and balanced score.

In addition, as contextual variables have a direct impact on player´s performances in racket sports [13], the current analyses controlled for total match duration, the influence of match set (1^st^, 2^nd^ or 3^rd^), set interval (before and after the regular time-out within each set), and score-line (point differences). These contextual variables from all matches were established as part of Stage 3 with clustering procedures run (two-step cluster analysis) to classify and redefine the variables of match type and score-line [13]. Further, the total match duration (minutes) was examined and classified matches into long and regular ones (see Table 1). The difference in the score between players was established as the score-line, which considered the influence of score (i.e., pressure) on the subsequent performances (long and immediate rally). Finally, the clustering analysis (see Table 1) identified 3 score-lines for both sexes: losing, balanced, or winning by 2 (male players) or 3 points (female players).

### Statistical analysis

Firstly, due to the use of match-contextual variables, the sample was split into different clusters that exemplified the specificities of competition (i.e. location and length of long rallies). Then, two-step cluster analyses were conducted separately for each sex including the following variables: set, interval, score-line and match type. This statistical model determined the best number of rally groups (clusters) using the Schwartz’s Bayesian Information Criterion (BIC = 2388.03 and 4377.71, for men´s and women´s rallies, respectively). Additionally, the log-likelihood distance measure was used to check for similarities between rally groups. The models obtained were good with Silhouette measures of 0.85 and 0.50, respectively, for men’s and women’s’ rallies. Accordingly, the sample was split into eight groups for men’s long rallies and three groups for women’s long rallies.

Secondly, the data normality for continuous variables was assessed using the Shapiro-Wilk and Kolmogorov-Smirnov’s tests (p<0.05). Only time between strokes during long rallies (both in men´s and women´s matches) displayed a normal distribution (p>0.05) and one-way ANOVA (Bonferroni´s post hoc test) examined time between strokes among clusters. Non-parametric tests compared performance differences between long and next rallies (repeated measures Wilcoxon test for rally time, rest time, density, number of strokes, and time between strokes) and performance differences among clusters (Kruskal Wallis test). The *r* effect size (ES) magnitude was calculated and interpreted as follows: small (0–0.30), moderate (0.31–0.50), or large (>0.50) [14].

Thirdly, the relationships between clusters obtained and rally outcome, and long and next rally variables and rally outcomes were determined via Pearson’s Chi-square tests (Crosstabs Command) with adjusted residuals (AR) > 1.96 considered as significant. In addition, Cramer’s *V* test was used to calculate ES with interpretations based on the following values: 0.10 = small effect, 0.30 = medium effect, and 0.50 = large effect [14]. All analyses were conducted using IBM SPSS for Windows version 23 (IBM. Corp. Armonk, NY) and significance level was set to 0.05.

## Results

Table 2 shows the results (distribution of rallies) of the two-step cluster analyses for men’s and women’s rallies. The clustering model identified eight different contexts (clusters) for long rallies during men’s matches. The most frequent context was cluster 5 (18.5%, during 1^st^ set of regular matches, after the interval and when losing), cluster 2 (15.7%, during 2^nd^ set of regular matches, after the interval and during balanced score-lines), cluster 1 (15.3%, during 1^st^ set of regular matches, before the interval and during balanced score-lines), cluster 3 (13.3%, during 3^rd^ set of long matches, after the interval and during balanced score-lines), cluster 8 (11.9%, during 1^st^ set of regular matches, after the interval and during balanced score-lines), and cluster 4 (10.8%, 1^st^ set of regular matches, before the interval and when winning). In addition, clusters 6 (7.2%, during 1^st^ set of long matches, before the interval and during balanced score-lines) and 7 (7.4%, during 3^rd^ set of long matches, before the interval and during balanced score-lines) were less frequent with these occurring specifically during long matches. During women’s matches, only three contexts (clusters) where long rallies happened were identified (Table 3). Cluster 3 was the most prevalent (38.7% during the 1^st^ set of long matches, before the interval and during balanced score-lines) while cluster 1 (33.3% during 3^rd^ set of regular matches, after the interval and during balanced score-lines) and 2 (28.0% during 1^st^ set of long matches, after the interval and when losing) were also characterised.

**Table 2:** Results of the long rally groups (clusters) identified by the two-step cluster analysis for men’s and women’s players (I = importance of variables when classifying long rallies; BIC = Schwartz’s Bayesian Information Criterion).

**Men**	**Cluster 1**	**Cluster 2**	**Cluster 3**	**Cluster 4**	**Cluster 5**	**Cluster 6**	**Cluster 7**	**Cluster 8**
N (%)	133 (15.3%)	136 (15.7%)	115 (13.3%)	94 (10.8%)	160 (18.5%)	62 (7.2%)	64 (7.4%)	103 (11.9%)
Set (I = 1.0)	1 (100%)	2 (100%)	3 (41.7%)	1 (53.0%)	1 (81.2%)	1 (100%)	3 (100%)	1 (100%)
Interval (I = 0.72)	Before (100%)	After (100%)	After (100%)	Before (100%)	After (100%)	Before (50.0%)	Before (100%)	After (100%)
Score-line (I = 0.53)	Balanced (100%)	Balanced (40.4%)	Balanced (45.2%)	Winning (55.3%)	Losing (45.0%)	Balanced (100%)	Balanced (75.0%)	Balanced (100%)
Match Type (I = 0.38)	Regular (66.9%)	Regular (100%)	Long (100%)	Regular (76.6%)	Regular (100%)	Long (100%)	Long (64.1%)	Regular (100%)
BIC	5806.54	4796.77	4074.58	3623.18	3241.31	2894.27	2630.48	2388.03
**Women**	**Cluster 1**		**Cluster 2**		**Cluster 3**			
N (%)	274 (33.3%)		230 (28.0%)		318 (38.7%)			
Set (I = 0.17)	3 (37.2%)		1 (46.1%)		1 (52.5%)			
Interval (I = 0.07)	After (54.0%)		After (70.4%)		Before (61.9%)			
Score-line (I = 1.0)	Balanced (46.4%)		Losing (47.4%)		Balanced (53.8%)			
Match type (I = 0.63)	Regular (100%)		Long (100%)		Long (100%)			
BIC	6099.37		5062.88		4337.71			

**Table 3:** Results of Kruskal Wallis (χ2) and one-way ANOVA (F-value) tests differentiating time-related factors among clusters for men´s and women´s long rallies.

Men´s clusters	χ2 or F-value	df	P-value	Pairwise comparisons
Number of strokes	13.17	7	0.07	---
Rally time	18.80	7	0.012	1vs3;1vs6;2vs6;3vs4;3vs5;3vs8;4vs6; 5vs6;6vs8
Rest time	64.11	7	<0.001	1vs3; 1vs7; 1vs8; 2vs3; 2vs7; 2vs8; 3vs4; 3vs5; 3vs6; 3vs8; 4vs5; 4vs6; 4vs7; 5vs7; 5vs8; 6vs8; 7vs8
Density	35.70	7	<0.001	1vs3; 1vs7; 1vs8; 2vs3; 2vs7; 2vs8; 3vs4; 3vs5; 3vs6; 3vs7; 3vs8; 4vs5; 4vs6; 4vs8; 5vs7; 5vs8; 6vs7; 7vs8
Time between strokes	2.58*	7	0.010	1vs3; 1vs6; 1vs7; 1vs8; 2vs3; 3vs4; 5vs6;
**Women´s clusters**				
Number of strokes	5.24	2	0.073	---
Rally time	12.33	2	0.002	1vs2; 1vs3
Rest time	100.7	2	<0.001	1vs2; 1vs3
Density	61.57	2	<0.001	1vs2; 1vs3
Time between strokes	7.23*	2	<0.001	1vs3

*F-value

Significant differences among clusters for rally time, rest time, density and time between strokes were identified for men’s and women’s long rallies (p<0.05; see Table 3).

The pairwise comparisons showed clear differences among clusters in men´s long rallies (see Table 5) with cluster 6 as the longest rally and clusters 1 and 8 as the shortest ones; clusters 3 and 7 were the ones with longer rest time and cluster 8 involved less rest time; clusters 4 and 8 were of higher density and clusters 3 and 7 were of lower density; and clusters 1 and 2 involved less time between strokes. For women´s long rallies, cluster 1 showed greater time between strokes, rally time and rest time, and higher density than clusters 2 and 3 (see Table 5).

No significant (p>0.05) relationships were identified among clusters for point outcome for both men’s and women´s matches (Table 4).

**Table 4:** Frequency distribution of point outcome for each cluster during men’s and women´s matches.

	Cluster 1			Cluster 2			Cluster 3			Cluster 4			Cluster 5			Cluster 6			Cluster 7			Cluster 8		
**Men**	N	%	AR	N	%	AR	N	%	AR	N	%	AR	N	%	AR	N	%	AR	N	%	AR	N	%	AR
Serving																								
Winner	26	19.5	0.0	20	14.7	-1.3	22	19.1	-0.2	23	24.5	1.6	43	26.9	1.7	5	8.1	-1.2	11	17.2	-0.8	18	17.5	-0.3
Forced error	26	19.5	2.0	16	11.8	0.1	12	10.4	-1.7	13	13.8	-0.4	19	11.9	-1.0	10	16.1	-0.5	8	12.5	0.0	20	19.4	1.5
Unforced error	25	18.8	0.1	24	17.6	-0.1	19	16.5	0.1	14	14.9	-0.3	30	18.8	2.0	6	9.7	-1.4	13	20.3	0.0	13	12.6	-1.2
Receiving																								
Winner	25	18.8	-0.3	30	22.1	0.3	27	23.5	1.5	15	16.0	-0.7	29	18.1	-0.1	8	12.9	-0.6	14	21.9	0.5	17	16.5	-0.8
Forced error	15	11.3	-0.8	25	18.4	1.9	18	15.7	-0.3	17	18.1	1.0	23	14.4	-1.3	11	17.7	0.6	5	7.8	-1.6	20	19.4	0.6
Unforced error	16	12.0	-1.0	21	15.4	-0.7	17	14.8	0.4	12	12.8	-1.3	16	10.0	-1.5	22	35.5	3.3	13	20.3	1.9	15	14.6	0.4
χ^2^	45.386																							
p	0.106																							
ES	0.21																							
	Cluster 1			Cluster 2			Cluster 3																	
**Women**	N	%	AR	N	%	AR	N	%	AR															
Serving																								
Winner	50	18.2	-0.8	58	25.2	1.7	56	17.6	-0.8															
Forced error	46	16.8	-0.1	47	20.4	1.4	48	15.1	-1.2															
Unforced error	36	13.1	-1.8	32	13.9	-1.5	72	22.6	3.1															
**Receiving**																								
Winner	55	20.1	1.8	30	13.0	-0.9	46	14.5	-0.9															
Forced error	44	16.1	-0.2	34	14.8	0.2	49	15.4	0.0															
Unforced error	43	15.7	1.2	29	12.6	-1.1	47	14.8	-0.2															
χ^2^	16.617																							
p	0.083																							
ES	0.13																							

During long rallies of men’s matches, rally time, rest time, density and time between strokes were significantly (all p<0.05) greater compared to the next rally for all clusters (Table 5). Similarly, the frequency of strokes was significantly greater during long rallies compared to the next rally but only for clusters 3, 5, 6 and 7 (all p<0.01, Table 5). Large effects (ES) were noted for rally time and rest time variables in clusters 2, 3, 5 and 8, and for density in clusters 2 and 8 (Table 5).

**Table 5:** Descriptive statistics (median and interquartile range) for time-related factors during long and next rallies during men’s matches.

	Long rally			Next rally				
		Quartile			Quartile			
Cluster 1	Median	Lower	Upper	Median	Lower	Upper	Z	ES
Number of strokes	20.00^c^	17.00	25.00	7.00	5.00	11.50	-7.81	-0.48
Rally time	18.18^c^	15.14	23.87	6.98	4.31	10.75	-7.55	-0.46
Rest time	26.94^c^	19.78	34.85	20.77	16.46	25.20	-3.61	-0.22
Density	0.75^c^	0.56	0.96	0.33	0.21	0.53	-7.64	-0.47
Time between strokes	0.91	0.85	0.96	0.90	0.83	0.97	-0.91	-0.06
Cluster 2								
Number of strokes	20.00^c^	17.00	26.00	8.00	5.00	14.00	-9.50	-0.58
Rally time	18.75^c^	15.65	24.68	7.31	4.23	12.37	-9.50	-0.58
Rest time	26.48^a^	19.17	34.97	20.60	15.56	30.73	-2.48	-0.15
Density	0.75^c^	0.55	0.97	0.34	0.22	0.51	-8.22	-0.50
Time between strokes	0.92	0.87	0.98	0.91	0.82	0.97	-1.57	-0.10
Cluster 3								
Number of strokes	21.00^c^	17.00	30.00	11.00	6.00	16.00	-8.11	-0.53
Rally time	19.23^c^	16.15	28.27	9.75	5.63	15.02	-8.05	-0.53
Rest time	33.28^c^	26.06	47.50	26.20	20.28	33.24	-4.15	-0.27
Density	0.62^c^	0.47	0.82	0.33	0.22	0.51	-6.95	-0.46
Time between strokes	0.95^c^	0.90	1.00	0.91	0.82	0.97	-3.76	-0.25
Cluster 4								
Number of strokes	19.50^c^	17.00	25.00	10.00	4.00	17.00	-6.60	-0.48
Rally time	18.18^c^	15.18	23.35	8.68	3.95	15.31	-6.54	-0.48
Rest time	25.61^c^	18.65	32.31	22.92	16.80	27.64	-2.69	-0.20
Density	0.81^c^	0.58	0.98	0.38	0.20	0.69	-5.91	-0.43
Time between strokes	0.94	0.89	0.99	0.91	0.83	0.99	-1.58	-0.12
Cluster 5								
Number of strokes	19.00^c^	17.00	27.00	8.00	4.00	13.75	-10.25	-0.57
Rally time	18.49^c^	15.21	24.87	7.11	3.99	12.19	-10.30	-0.58
Rest time	27.08^c^	21.70	37.55	18.36	14.56	24.56	-6.28	-0.35
Density	0.69^c^	0.49	0.94	0.35	0.23	0.52	-8.54	-0.48
Time between strokes	0.92^c^	0.88	0.97	0.89	0.82	0.97	-3.48	-0.19
Cluster 6								
Number of strokes	23.00^c^	18.00	30.00	9.50	6.00	17.00	-5.19	-0.47
Rally time	20.91^c^	16.21	28.59	8.91	5.73	16.87	-5.17	-0.46
Rest time	28.98^b^	23.12	40.68	21.60	16.37	30.52	-2.60	-0.23
Density	0.73^c^	0.58	1.04	0.40	0.23	0.56	-4.36	-0.39
Time between strokes	0.95^b^	0.90	0.99	0.90	0.85	0.97	-3.12	-0.28
Cluster 7								
Number of strokes	21.00^c^	17.00	25.00	8.50	5.00	14.75	-5.77	-0.51
Rally time	20.49^c^	15.38	23.88	6.95	4.75	13.45	-5.71	-0.50
Rest time	32.64^c^	25.13	47.24	24.24	17.92	29.88	-4.41	-0.39
Density	0.60^c^	0.49	0.79	0.30	0.18	0.57	-4.58	-0.40
Time between strokes	0.96^b^	0.90	1.00	0.93	0.78	0.98	-2.86	-0.25
Cluster 8								
Number of strokes	18.00^c^	16.00	24.00	8.00	4.00	14.00	-7.46	-0.52
Rally time	17.62^c^	15.07	22.00	7.70	3.58	13.06	-7.42	-0.52
Rest time	22.84^a^	17.40	27.99	17.35	14.85	26.74	-2.14	-0.15
Density	0.82^c^	0.67	1.14	0.37	0.21	0.67	-7.51	-0.52
Time between strokes	0.94	0.89	0.98	0.91	0.84	0.97	-1.87	-0.13

^a^: p<0.05,

^b^: p<0.01,

^c^: p<0.001 vs. Next rally; ES: effect size

During long rallies of women’s matches, rally time, rest time, density, and time between strokes were significantly (all p<0.05) greater compared to the next rally for all clusters (Table 6). However, the time between strokes was significantly lower during long rallies compared to the next rally for all clusters (all p<0.05, Table 6). Large effects (ES) were noted for rally time, rest time, and density variables in all clusters (Table 6).

**Table 6:** Descriptive statistics (median and interquartile range) for time-related factors during long and next rallies during women’s matches.

	Long rally			Next rally				
		Quartile			Quartile			
Cluster 1	Median	Lower	Upper	Median	Lower	Upper	Z	ES
Number of strokes	18.00^c^	15.00	23.00	7.00	4.00	12.00	-12.46	-0.53
Rally time	18.80^c^	15.27	23.77	7.55	4.23	12.34	-12.77	-0.54
Rest time	31.30^c^	25.62	41.57	25.44	21.15	34.63	-4.62	-0.19
Density	0.61^c^	0.49	0.74	0.28	0.18	0.44	-11.79	-0.50
Time between strokes	1.02^a^	0.95	1.08	1.03	0.94	1.16	2.56	0.10
Cluster 2								
Number of strokes	18.00^c^	15.00	21.00	6.00	4.00	12.00	-11.81	-0.55
Rally time	17.90^c^	15.08	21.70	6.63	4.33	11.97	-11.73	-0.54
Rest time	24.80^c^	19.97	29.90	19.82	15.55	25.68	-5.52	-0.25
Density	0.76^c^	0.60	0.95	0.35	0.22	0.52	-10.86	-0.50
Time between strokes	1.00^b^	0.93	1.06	1.00	0.90	1.15	2.65	0.12
Cluster 3								
Number of strokes	17.00^c^	15.00	21.00	6.00	4.00	10.00	-14.33	-0.56
Rally time	17.16^c^	15.00	20.40	6.48	4.20	9.70	-14.17	-0.56
Rest time	24.14^c^	19.29	29.87	20.00	16.98	26.24	-5.65	-0.22
Density	0.75^c^	0.60	0.92	0.32	0.21	0.49	-13.62	-0.54
Time between strokes	0.99^a^	0.91	1.05	0.99	0.89	1.13	2.04	0.08

^a^: p<0.05,

^b^: p<0.01,

^c^: p<0.001 vs. Next rally; ES: effect size

Significant relationships between rallies and point outcome for men’s matches were identified (Table 7) for two specific match contexts: (i) during cluster 5 (small ES = 0.22) with more points won by the serving player during long rallies compared to the next rallies (26.3% vs. 15.0%; AR = 2.5), and with more points won via unforced errors when serving during the next rallies compared to the long rallies (23.1% vs. 10.0%; AR = 3.2); and (ii) during cluster 6 (moderate ES = 0.35) with more points won by the receiving player during the next rallies compared to the long rallies (25.8% vs. 8.1%; AR = 2.6), and with more points won via unforced errors when receiving during long rallies compared to next rallies (33.9% vs. 17.7%; AR = 2.1).

**Table 7:** Frequency distribution of point outcome for long rally and the next rally according to each cluster during men’s matches.

Point Outcome	Cluster	Long rally			Next rally					
	Cluster 1	N	%	AR	N	%	AR	*χ*^2^	p	ES
Serving	Winner	27	20.3	0.3	25	18.8	-0.3			
	Forced Error	27	20.3	0.0	27	20.3	0.0	4.66	0.458	0.13
	Unforced Error	23	17.3	1.6	14	10.5	-1.6			
Receiving	Winner	26	19.5	0.3	24	18.0	-0.3			
	Forced Error	14	10.5	-1.1	20	15.0	1.1			
	Unforced Error	16	12.0	-1.2	23	17.3	1.2			
	Cluster 2									
Serving	Winner	20	14.7	-1.7	31	22.8	1.7			
	Forced Error	16	11.8	-0.9	21	15.4	0.9	7.163	0.210	0.16
	Unforced Error	25	18.4	0.6	21	15.4	-0.6			
Receiving	Winner	29	21.3	0.3	27	19.9	-0.3			
	Forced Error	26	19.1	2.1	14	10.3	-2.1			
	Unforced Error	20	14.7	-0.3	22	16.2	0.3			
	Cluster 3									
Serving	Winner	22	19.1	1.5	14	12.2	-1.5			
	Forced Error	12	10.4	-0.6	15	13.0	0.6	8.071	0.154	0.19
	Unforced Error	18	15.7	0.8	14	12.2	-0.8			
Receiving	Winner	27	23.5	-0.9	33	28.7	0.9			
	Forced Error	18	15.7	1.6	10	8.7	-1.6			
	Unforced Error	18	15.7	-1.8	29	25.2	1.8			
	Cluster 4									
Serving	Winner	23	24.5	0.9	18	19.1	-0.9			
	Forced Error	12	12.8	-0.6	15	16.0	0.6	3.838	0.573	0.14
	Unforced Error	15	16.0	-0.2	16	17.0	0.2			
Receiving	Winner	15	16.0	-0.9	20	21.3	0.9			
	Forced Error	17	18.1	1.5	10	10.6	-1.5			
	Unforced Error	12	12.8	-0.6	15	16.0	0.6			
	Cluster 5									
Serving	Winner	42	26.3	2.5	24	15.0	-2.5			
	Forced Error	20	12.5	-0.5	23	14.4	0.5	15.279	0.008	0.22
	Unforced Error	31	19.4	-0.3	33	20.6	0.3			
Receiving	Winner	29	18.1	0.0	29	18.1	0.0			
	Forced Error	22	13.8	1.4	14	8.8	-1.4			
	Unforced Error	16	10.0	-3.2	37	23.1	3.2			
	Cluster 6									
Serving	Winner	5	8.1	-2.6	16	25.8	2.6			
	Forced Error	9	14.5	1.5	4	6.5	-1.5	15.537	0.009	0.35
	Unforced Error	6	9.7	-1.5	12	19.4	1.5			
Receiving	Winner	9	14.5	-0.9	13	21.0	0.9			
	Forced Error	12	19.4	1.5	6	9.7	-1.5			
	Unforced Error	21	33.9	2.1	11	17.7	-2.1			
	Cluster 7									
Serving	Winner	11	17.2	-0.2	12	18.8	0.2			
	Forced Error	8	12.5	-1.0	12	18.8	1.0	5.391	0.381	0.21
	Unforced Error	13	20.3	2.0	5	7.8	-2.0			
Receiving	Winner	14	21.9	-0.4	16	25.0	0.4			
	Forced Error	5	7.8	-0.9	8	12.5	0.9			
	Unforced Error	13	20.3	0.5	11	17.2	-0.5			
	Cluster 8									
Serving	Winner	18	17.5	-0.2	19	18.4	0.2			
	Forced Error	20	19.4	1.3	13	12.6	-1.3	4.321	0.509	0.15
	Unforced Error	13	12.6	-1.0	18	17.5	1.0			
Receiving	Winner	16	15.5	0.2	15	14.6	-0.2			
	Forced Error	20	19.4	0.9	15	14.6	-0.9			
	Unforced Error	16	15.5	-1.2	23	22.3	1.2			

AR = adjusted residuals. ES = effect size; χ^2^ = Pearson’s Chi-square.

Significant relationships between rallies and point outcome for women’s matches were identified (Table 8) during cluster 2 (small ES = 0.18) with more points won for the serving player during long rallies compared to the next rallies (25.2% vs. 17.0%; AR = 2.2), and with more points won via unforced errors when receiving during the next rallies compared to long rallies (22.2% vs. 12.6%; AR = 2.7).

**Table 8:** Frequency distribution of point outcome for long rally and the next rally according to each cluster in women’s matches.

Point Outcome	Cluster	Long rally			Immediate next rally					
	Cluster 1	N	%	AR	N	%	AR	χ^2^	p	ES
Serving	Winner	50	18.2%	1.3	39	14.2%	-1.3			
	Forced Error	46	16.8%	0.6	41	15.0%	-0.6			
	Unforced Error	36	13.1%	-0.1	37	13.5%	0.1	9.767	0.073	0.13
Receiving	Winner	55	20.1%	-0.8	63	23.0%	0.8			
	Forced Error	44	16.1%	1.9	29	10.6%	-1.9			
	Unforced Error	43	15.7%	-2.4	65	23.7%	2.4			
	Cluster 2									
Serving	Winner	58	25.2%	2.2	39	17.0%	-2.2			
	Forced Error	47	20.4%	1.3	36	15.7%	-1.3			
	Unforced Error	32	13.9%	-0.8	38	16.5%	0.8	14.239	0.012*	0.18
Receiving	Winner	30	13.0%	-1.3	40	17.4%	1.3			
	Forced Error	34	14.8%	1.1	26	11.3%	-1.1			
	Unforced Error	29	12.6%	-2.7	51	22.2%	2.7			
	Cluster 3									
Serving	Winner	56	17.6%	0.5	51	16.0%	-0.5			
	Forced Error	48	15.1%	-0.4	52	16.4%	0.4			
	Unforced Error	72	22.6%	1.8	54	17.0%	-1.8	8.769	0.121	0.12
Receiving	Winner	46	14.5%	-0.8	53	16.7%	0.8			
	Forced Error	49	15.4%	1.1	39	12.3%	-1.1			
	Unforced Error	47	14.8%	-2.3	69	21.7%	2.3			

AR = adjusted residuals; ES: effect size size; χ^2^ = Pearson’s Chi-square.

## Discussion

The aim of the current study was two-fold: (i) to identify the contexts where long rallies occur while differentiating time-related and technical parameters; and (ii) to identify performance differences between long rallies and the subsequent rally played according to the match-context and players’ sex. The current findings highlighted the importance of separating analyses by sex and the need to consider the specificities of match-contexts. In particular, men and women’s matches exhibited different long rally contexts with eight and three clusters, respectively [10]. Specifically, clear differences among clusters were identified for time-related parameters (i.e., rally and rest time, density and time between strokes) indicating the specific and independent scenarios where long rallies occur based on contextual variables. In addition, different performances were noted during long and the immediate next rallies from time-related (i.e., rally time, rest time, strokes per rally, density, and time between strokes) and technical (i.e., different point outcome after long rally for men’s players in clusters 5 and 6, and for women’s players in cluster 2) points of view. Therefore, our results confirm our hypothesis that rallies played immediately after a long rally involve lower time-related parameters and more unforced errors modulated by several contextual factors.

One intriguing finding of the current study was the distribution of total long rallies in men and women’s matches. The frequency of these high-demanding rallies was 24.5% during men’s matches and 19.6% during women’s matches, values greater than the 15% reported by Laffaye et al. [3] during the London 2012 Olympic Games. Our results confirm the current trends of the intermittent and high intensity demands required by players during elite badminton matches and the increasing number of long rallies played during matches by both sexes [10]. Further, the current findings corroborate that elite badminton matches mainly consist of short-intermittent rallies (around 75% and 80% of men’s and women’s matches, respectively) interspersed with long rallies that modify players’ demands [3–5]. Further, this finding points out the necessity in performance analyses to scrutinize published reports with contemporary data as sports like badminton constantly evolve with different demands exhibited by players that need to be taken into account during training and coaching practices.

Furthermore, the contexts (clusters) in which the long rallies occurred differed according to sex and match-contextual variables, reflecting the importance of match scenarios that modify players’ responses and their subsequent performances [10,13]. In fact, male players experienced a greater variability of contexts where long rallies occurred (clusters 1, 2, 4, 5 and 8 during 1^st^ and 2^nd^ sets of regular matches; and clusters 3, 6 and 7 during 1^st^ and 3^rd^ sets of long matches) compared to female players who experienced only three main contexts, two during long matches (clusters 2 and 3 during 1^st^ set) and one during regular matches (cluster 1 during 3^rd^ set). These findings reflected that clusters were different for time-related parameters (i.e., rally time, rest time, density and time between strokes) with each context distinctively affecting player´s performance. Abdullahi et al. [2] recommended that coaches and players should focus on these time-related performance indicators as evidence-based guidelines for training and match preparation. Therefore, our results provide further support that consideration of these match-context constraints is extremely important to understand the differences between long rallies and immediate next rallies from a time-related and technical analysis approach [3–4,15]. Consideration of these contextual influences would allow modelling of match perturbations or critical incidents to identify potential player performance decrements [16] and training strategies to enhance players’ focus during different match stages (intervals, sets) that are comparable to real scenarios [17].

For men’s matches, significantly greater rally time, rest time, density, and strokes per rally were identified during long rallies compared to the next rally for all clusters. Additionally, the time between strokes was higher during long rallies (i.e. increased intensity with shorter time between strokes) compared to the next rally for clusters 3, 5, 6, and 7. These findings may support Liddle and O’Donoghue’s [7] conclusions that rallies following a long rally were shorter and less intense as a result of physical and mental fatigue after the prior long effort. Subsequently, players may feel more fatigued due to the high-intensity effort during a long rally and undertake more rest time to recover for the next rally. This may also be a critical time of the match and therefore players need to be well prepared for these kinds of situations [7].

For women’s matches, a divergent result was observed with long rallies consisting of less time between strokes for all match contexts (clusters 1, 2, and 3) despite greater rally time, rest time, density, and strokes per rally. This result may indicate that female players increase the intensity of play immediately after a long rally, trying to take advantage of the potential fatigue produced during the long rally. Further, it may also indicate that female players pace themselves during long rallies to minimise physical fatigue [7] and thereby might have more reserves to maintain the technical, tactical, physical, and psychological aspects for matches [16]. In particular, the combination of accumulative physical and mental fatigue may produce different tactics from players during each competition scenario including long and immediate next rallies to minimise debilitating performances (e.g., higher time between shots after long rallies) as the set/match goes on [9]. On the other hand, the relationship between clusters and point outcome was not significant. This may reflect that players perform with different physical intensities (i.e., time-related factors) during long rallies that does not directly affect point outcome. However, some differences were identified when accounting for context variables in assessing the relationship between long rally and the immediate next rally. In particular, only two specific contexts during men’s matches (clusters 5 and 6) and only one context during women’s matches (cluster 2) were significant. These findings highlight the importance of controlling for contextual variables as these have the potential to modify time-related factors of long rallies during men´s (balanced and losing score-lines during both intervals in regular and long matches) and women´s (interval of 11 to 21 points when losing and playing long matches) matches [10,13]. Further, this indirectly demonstrates the importance of simulating specific match scenarios in practice or creating certain scenarios within a match (e.g., forcing opponent’s errors after a stressful/long rally). Liddle and O’Donoghue [7] reported that more unforced errors occurred due to physical and mental fatigue during next rallies after long rallies. Results for men’s cluster 5 and women’s cluster 2, where players won more points when serving during the long rally but lost the immediate next rally via an unforced error, provided further support of this fatigue-induced reasoning. More specifically, this pattern of results might have been induced by physical fatigue during high-competitive conditions [8–9] which in turn impacted on players’ cognitive performance during and after long rallies (e.g., visual search, anticipation or decision-making selecting the correct technique/ response). However, the opposite pattern occurred within men’s cluster 6 (with the longest rally time of all clusters) where players won long rallies via the opponent’s unforced errors and then won the immediate next rally via a clear winning shot. In this context, male players may have taken advantage of their opponent’s early 1^st^ set mistakes and fatigue-onset during high-demanding, long rallies (e.g. balanced score-lines of long matches) by attacking the immediate next point due to their own reduced physical/cognitive fatigue (i.e. better physical/cognitive fitness). These results clearly highlight the importance of analysing the physical and psychological demands that players experience during different scenarios (i.e., clusters) of the set and/or match [9].

The current results are helpful in developing specific practical applications for coaches and athletes in both training interventions and match tactics and strategies [18]. Specifically, for women’s matches, we would recommend that players undertake simulated intermittent activity that replicates long and immediate next rallies during the 3^rd^ set of regular matches (dealing with winning and close score-lines) and 1^st^ set of long matches (both losing and winning score-lines). Such training simulations would better prepare players physiologically and cognitively for these competitive scenarios during matches. For men’s matches, we recommend that players consider the importance of higher frequencies of strokes during long rallies with match contexts similar to clusters 3, 5, 6, and 7. Specifically, training drills should control for fatigue effects in the immediate next rally during simulations of the 1^st^ set of regular matches (losing during the interval of points 11 to 21) and long matches (with balanced score-lines and interval of points from 0 to 11). The use of a minimum number of strokes and range of rally durations (under fatigue conditions) would help better prepare players so that they perform with less unforced errors during and after long rallies.

To our knowledge, the current study was the first to explore time-related and technical demands of long rallies and their immediate impact on the next rally in elite badminton while considering contextual factors. However, some limitations of the present research need to be considered for further studies. Firstly, the variables examined in this study were limited to those reported in previous studies, while future studies might include further technical (e.g., technical actions used during both rallies), tactical (e.g., zones of the court used to start and end both rallies), time-related (e.g., accumulated playing time when the long rally occurs), and match-contextual (i.e., quality of opposition) variables. Secondly, the examination of matches during consecutive tournaments (e.g. World Championships or Olympic Games) may produce different performance trends during long and immediate next rallies, and, hence, should be considered in forthcoming research. Thirdly, matches were randomly selected from major tournaments and included both regular/group and knockout/elimination matches. As only one study investigated the differences between stages [4] this study was a preliminary investigation with future studies of distinct stages recommended. Additionally, future studies might want to adopt a multivariate approach to define the match-context and performance influences during long rallies in elite badminton to assist coaches with player preparation [18–19].

In conclusion, the current study identified and characterised long rallies in elite men´s and women´s badminton matches highlighting the importance of sex and contextual factors on time-related and technical demands. The results identified eight and three different contexts of long rallies for men and women’s matches, respectively. Specifically, men´s clusters 1 and 2 (longer time between strokes), 6 (longer rally time), 7 (longer rest time) and 8 (shorter rally time); and women´s cluster 1 (longer rally time and rest time, and longer time between strokes) demonstrated clear and different time-related demands than the other clusters for their sex. Further, different time-related factors and point outcome of long rallies and the immediate next rally were identified for both sexes. In particular, different time between strokes were identified during next rallies compared to long rallies that varied with match context (e.g. longer in women’s matches after the long rally and shorter in men’s matches after long rallies). Additionally, the point outcome was associated with an increased number of unforced errors after playing long rallies in men’s cluster 5 and women’s cluster 2. This research knowledge is essential and innovative in order to have a better understanding of the time-related and technical demands of elite badminton where intermittent and different critical scenarios occur [4,15]. The information obtained from these sequences of play (i.e., long and immediate next rallies) will assist coaches in their modelling and simulation of players’ performances (i.e., physiologically and cognitively) during these unique match contexts [3,18].

## Supporting information

S1 Dataset IBM SPSS dataset of men´s badminton players. (SAV)

S2 Dataset IBM SPSS dataset of women´s badminton players. (SAV)
